# Antitumor Activity of PEGylated and TEGylated Phenothiazine Derivatives: Structure–Activity Relationship

**DOI:** 10.3390/ijms24065449

**Published:** 2023-03-13

**Authors:** Sandu Cibotaru, Andreea-Isabela Sandu, Alina Nicolescu, Luminita Marin

**Affiliations:** “Petru Poni” Institute of Macromolecular Chemistry, 700487 Iasi, Romania

**Keywords:** phenothiazine, poly(ethylene glycol), imines, transimination, anticancer, farnesyltransferase

## Abstract

The paper aims to investigate the antitumor activity of a series of phenothiazine derivatives in order to establish a structure–antitumor activity relationship. To this end, PEGylated and TEGylated phenothiazine have been functionalized with formyl units and further with sulfonamide units via dynamic imine bonds. Their antitumor activity was monitored in vitro against seven human tumors cell lines and a mouse one compared to a human normal cell line by MTS assay. In order to find the potential influence of different building blocks on antitumor activity, the antioxidant activity, the ability to inhibit farnesyltransferase and the capacity to bind amino acids relevant for tumor cell growth were investigated as well. It was established that different building blocks conferred different functionalities, inducing specific antitumor activity against the tumor cells.

## 1. Introduction

Cancer is the second leading cause of death in recent decades and is predicted to have a fast-growing incidence and mortality rate in the next 20 years [1]. While localized tumors can be cured with a high success rate by surgery or radiation therapy, the metastasis of the cells from primary tumors to unknown locations restrict the treatment to the systematic administration of antitumor drugs (i.e., chemotherapy). Despite progress in the research of cancer treatments, chemotherapy remains the main remedy of patients with metastatic cancer [2]. Unfortunately, its success rate is low (the 5-year relative survival rate for patients with distant metastasis is lower than 30% [3]), on one hand because of nonselective mechanism of action of the cancer drugs resulting in significant toxic effects to noncancerous tissues with important long term side effects and on the other hand because many drugs that are currently in clinical practice have multidrug resistance [4,5]. In this context there is a great demand for improving cancer chemotherapy, and the researcher’s efforts towards this aim revealed the development of novel multifunctional anticancer drugs that combine two or more pharmacophores in a single molecule as the future of chemotherapy [6]. In this regard, many pharmacophores combinations based on inorganic [7,8,9] and/or organic compounds [10,11,12,13,14,15] are currently investigated. Among the organic pharmacophores, nitrogen-containing heterocycles proved to have the most promising anticancer activity, being studied in various combinations [16,17,18]. Nevertheless, the investigation of phenothiazine compounds as anticancer drugs is still in its infancy [19]. Phenothiazine is a heterocyclic unit used in the synthesis of a large realm of drugs with various activities, being considered one of the most versatile heterocycles from the view of biological activity [20]. While predominantly used in psychopharmacology, recent studies revealed the important role of phenothiazine in cancer research [21,22]. Investigations of antipsychotic phenothiazine drugs approved by the FDA, such as fluphenazine, perphenazine, thioridazine and prochlorperazine, revealed anticancer activity against different tumor cell lines, such as glioblastoma, leukemia, and breast, colorectal and liver cancer [22]. Deeper investigations revealed that the antitumor mechanism of phenothiazine is correlated with its ability to alter the production of reactive organic species (ROS) or direct inhibition of multidrug resistance-associated proteins [22]. In addition, it was suggested that due to the sedative and antiemetic properties, the phenothiazine compounds can have palliative outcome on chemotherapy side effects [23]. These data encouraged further research for the improvement of anticancer activity by structural modifications [24] with various heteroaromatic units [25,26,27], leading to promising results, with an in vivo tumor inhibition of approx. 70% being reported [28]. Their deeper investigation brought into researcher attention another potential anticancer mechanism, that is, inhibition of enzymes with a key role in tumor cell proliferation, such as farnesyltransferase [26,29,30,31].

An important building block in the design of anticancer drugs is polyethylene glycol (PEG). PEG is a synthetic biocompatible polymer approved by the FDA for in vivo bioapplications [28]. PEG is highly hydrophilic and has no interaction with blood components, being used for the development of amphiphilic architectures for drug release [32], many of them being already FDA approved [https://www.biochempeg.com/article/58.html] (accessed on 3 January 2023). Recent investigations demonstrated that PEG significantly increases the pharmaceutical value of antitumor drugs by increasing the half-life and reducing the dose frequency due to its ability to enhance the retention time due to protection against various degradation mechanisms that are active inside tissues and cells [33].

Another important building block in the drug design is sulfonamide, the chemical motif of sulphonamides, a class of drugs with clinical use as antibacterial, diuretic, hypoglycemic and antithyroid agents [34]. Recent investigations revealed that the sulfonamide group promotes antitumor activity by triggering a variety of mechanisms, with a role in inhibition of tumor cell proliferation [35,36,37,38,39].

**Scheme 1:**
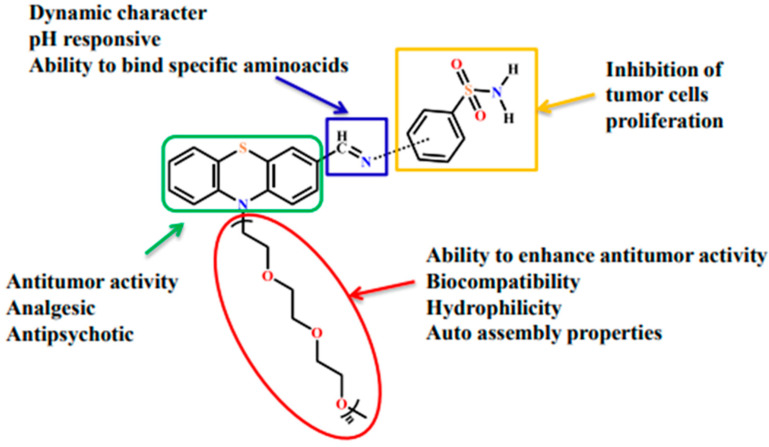
The rational design of the studied phenothiazine imines.

This literature survey, cumulated with our own research in the phenothiazine field [29,40,41,42], encouraged us to design new structures of antitumor drugs based on phenothiazine, PEG and sulfonamide units. Imine bond, known for its reversible formation leading to dynamic systems prone to respond under external stimuli [43,44,45], was used as a linking unit (Scheme 1). This choice took also into consideration that the pH responsive nature of the imine units to the medium acidosis of tumor tissues can trigger the controlled release of the antitumor building block [46] and can facilitate the binding of amino acids necessary for survival and proliferation of cancer cells [47].

## 2. Results and Discussion

### 2.1. The Structure and Rational Design

Two formyl phenothiazine derivatives substituted with tri(ethylene glycol) and poly(ethylene glycol), respectively, were reacted with two aromatic amines bearing a sulfonamide unit to form four imine derivatives (Scheme 1), and their antitumor activity was investigated in vitro on eight tumor lines. The rational design of the compounds emerged from our last research studies combined with literature data. The design contains building blocks with complementary activities meant to maximize selective antitumor activity and minimize the systemic side effects associated with chemotherapy. Thus, (i) phenothiazine was chosen for its selective efficiency against tumor cells and its analgesic and antipsychotic activity, which can overcome the side effects of chemotherapy such as nausea and vomiting; (ii) PEG has the ability to improve anticancer activity against tumor cells while being biocompatible, (iii) the sulfonamide unit is an adjuvant for improving antitumor activity and (iv) dynamic imine units are pH-responsive, having the potential to inhibit amino acids and an essential role in tumor cell metabolism.

### 2.2. In Vitro Investigation of the Antitumor Activity

**Figure 1:**
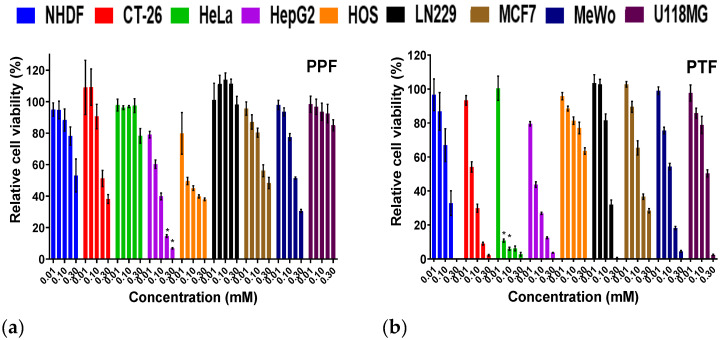

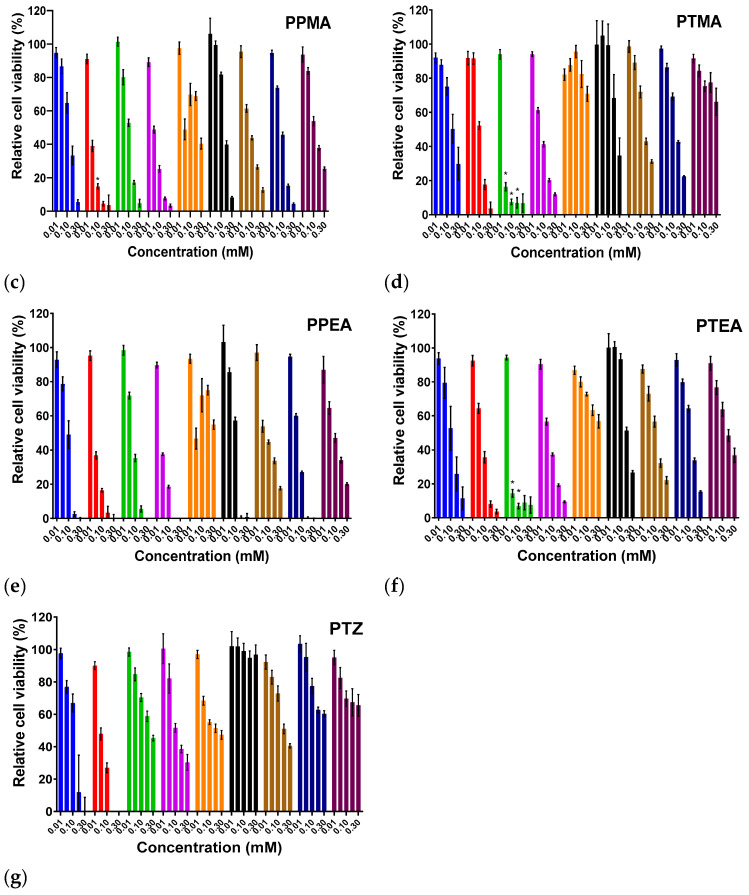
Graphical representation of the relative cell viability of normal fibroblasts, different human tumor cell lines and CT26 mouse tumor cell line when in contact with different concentrations of PTF, PPF, PTMA, PTEA, PPMA, PPEA and PTZ, * *p* < 0.05 for NHDF vs. tumor cell lines by two-way ANOVA.

**Scheme 2:**
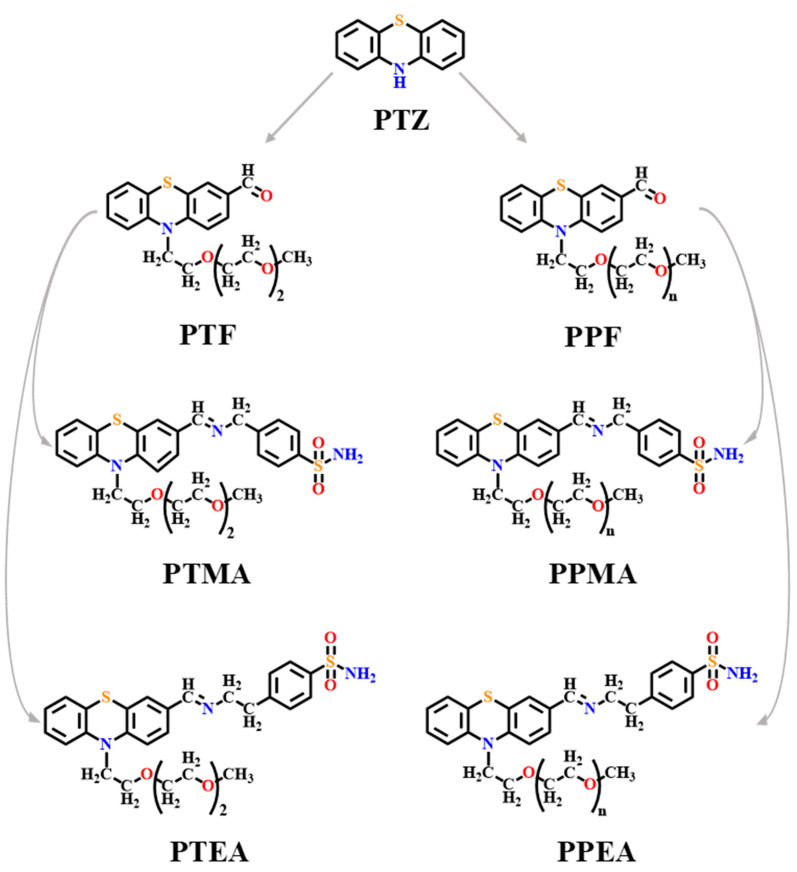
Structure of the studied compounds and their codes.

The antitumor activity of the synthesized imines (PPMA, PPEA, PTMA, PTEA) and their precursors (PPF, PTF and PTZ) (Scheme 2 and Scheme S1) was investigated by cytotoxicity determination on seven human cancer cell lines: cervical carcinoma (HeLa), malignant melanoma (MeWo), osteosarcoma (HOS), breast cancer (MCF7), liver cancer (HepG2), glioblastoma (LN229) and glioblastoma grade IV (U18MG), a mouse colon carcinoma cell line (CT26) and a normal cell line (NHDF). The cytotoxicity tests were done for solutions with concentration from 0.01 mM up to 0.3 mM, and the IC_50_ was determined for each cell line. The obtained results are graphically represented in Figure 1.

**Figure 2:**
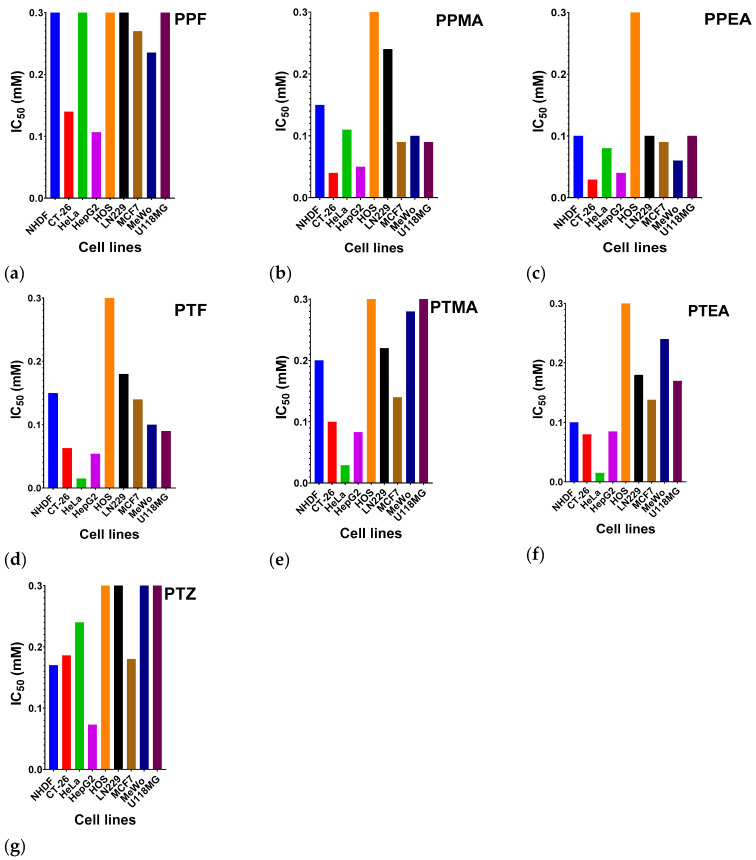
Graphical representation of the IC_50_ parameter.

The analysis of the compound’s cytotoxicity showed a relationship between the chemical structure and the cytotoxic effect. At first glance, it was obvious that the presence of a PEG or TEG chain and a formyl group on the phenothiazine core influenced the cytotoxic effect. Thus, the TEGylated aldehyde (PTF) showed an increased cytotoxic activity against colon carcinoma (CT-26), cervical carcinoma (HeLa) and liver cancer (HepG2), the best activity and selectivity being noticed on HeLa cells, with a cell viability of 11% at a concentration of 0.05 mM, compared to 86% on normal cells (NHDF) at the same concentration (Figure 1b). On CT-26 and HepG2, the cell viability of PTF was 30% and 27%, respectively, at a concentration of 0.1 mM. The IC_50_ parameter calculated for PTF on the tested cell lines confirmed the observation made on relative viability graphs, the best results being obtained for CT-26 (63 µM), HepG2 (52 µM) and HeLa (15 µM) (Figure 2d, Table S1). Also, the selectivity index calculated for the cell lines with relevant IC_50_ values indicated the best value as SI = 10 for HeLa (Table S2).

**Figure 3:**
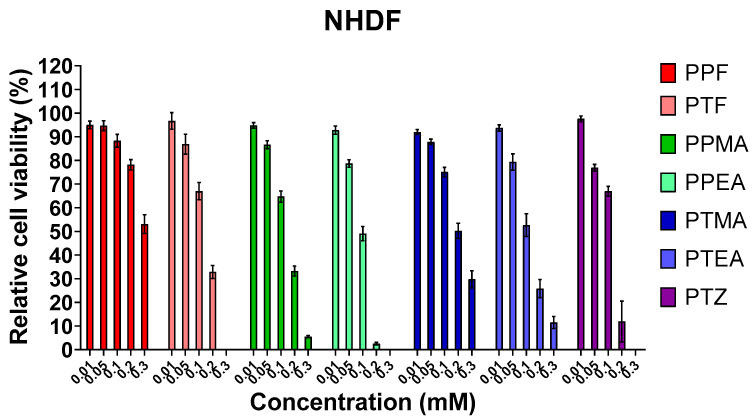
Cytotoxicity (MTS) assay: graphical representation of metabolic activity of NHDF cell line after being in contact 48 h with studied compounds in various concentrations. The results are presented as a mean value ± the standard error of the mean (S.E.M.), n = 7. No significant statistic differences were observed.

It was observed that the poly(ethylene glycol) chain improved more the biocompatibility on normal cells compared to tri(ethylene glycol) (Figure 3); that is, increasing the concentration of the tested compounds to 0.2 mM, the NHDF viability of PPF diminished only to 78%, while that of PPT diminished to 33% (Figure 1a). Nevertheless, the biocompatibility on the cancer cells improved too; by comparing the data obtained for PPF with those of PTF and phenothiazine (PTZ) used as reference, its cytotoxic effect was lower for all cell lines (Figure 1a,b,g and Figure 2a,d,g). However, on HepG2, PPF showed very good results, with a cell viability of 15% being recorded at a concentration of 0.2 mM. The influence of poly(ethylene glycol) length on biocompatibility improvement was confirmed also by the high values of IC_50_ calculated for PPF in contact with the tumor cells except CT-26 and HepG2, which gave lower values of 0.14 mM and 0.10 mM, respectively. It is worth mentioning that PTF displayed high specificity and selectivity on HeLa cells compared to its counterpart PPF. All in all, it can be concluded that the introduction of a short PEG chain, that is, tri(ethylene glycol), provided a good balance between the biocompatibility on normal cells and cytotoxic effect on tumor cells.

For imine compounds, a different behavior was noticed. The results obtained for the TEGylated imines did not show improvement in the cytotoxic effect compared to the parent aldehyde except for HeLa cells, which gave similar cell viability around 15% at a concentration of 0.05 mM. However, even if the IC_50_ values calculated for HeLa cells were also maintained at low values (0.029 mM and 0.015 mM, respectively), the selectivity index revealed a lower value than TEGylated aldehyde (~6.7 compared to 10) (Table S2). This unexpected diminishing of the antitumor effect can be attributed to the lower solubility of these compounds. The solubility of the TEGylated imines was significantly lower compared to the PEGylated ones; they partially precipitated in the culture media, and thus their lower antitumor activity could be assigned to their lower bioavailability. By analyzing the relative viability graphs of PTMA and PTEA compared to PTF, some differences can be observed (Figure 1d,f and Figure 3). The introduction of sulfonamide moiety increased the NHDF viability, especially in the case of PTMA, the PTEA being more cytotoxic. The same variation was observed for all cell lines, the cytotoxicity being higher in the case of PTEA compared to PTMA. 

The PEGylated compounds have a more pronounced cytotoxic effect in comparison with TEGylated ones, especially on CT-26, HepG2, LN229 and MCF7 confirmed by low values of IC50; that is, for CT-26, 0.04 mM (PPMA) and 0.029 mM (PPEA), and for HepG2, 0.054 mM (PPMA) and 0.04 mM (PPEA). Nevertheless, they also were cytotoxic for normal cells (NHDF), indicating low selectivity for cancer cells. The increased cytotoxic effect can be a consequence of increased solubility induced by the PEG chain and therefore of bioavailability. Similar to TEGylated imines, the PPEA showed a slightly higher cytotoxic effect compared to PPMA, suggesting that the higher number of methylene groups between phenothiazine and sulfonamide units leads to cytotoxicity increase. Analyzing all the data, it can be concluded that TEGylated derivatives have a greater affinity for HeLa cells, showing good selectivity at lower concentration. The specificity for HeLa cells appeared to be improved by the presence of a sulfonamide unit and an ethylene unit as well, an effect also obvious for CT26 and HepG2 lines. The introduction of the PEG chains improved the solubility and consequently the bioavailability, which was reflected in the improvement of the cytotoxicity against tumor lines especially in the case of the imine derivatives.

Moreover, comparing the obtained results with those reported for some traditional antitumor drugs (Table S3), it can be seen that even the IC_50_ values of the studied compounds were higher and the selectivity index in almost all cases was better, highlighting the potential of the new proposed design for developing new antitumor drugs. Unfortunately, among the antitumor phenothiazine derivatives reported in the literature, no cytotoxicity data on normal cell lines were found, making it difficult to do proper comparisons (Table S4). Nevertheless, compared to the phenothiazine reference, it is clear that the PEGylation of phenothiazine is a suitable pathway for improving the selectivity index towards more efficient drugs with fewer side effects.

### 2.3. In Vitro Radical Scavenging Activity

Literature data indicate the ability of phenothiazine to inhibit ROS as an aiding factor for antitumor activity improvement. This was attributed to the presence of heteroatoms in its structure [48], and it was demonstrated on its derivatives such as thioridazine, trifluperazine, chlorpromazine, promethazine and levopromazine [49]. The ROS production starts at the mitochondrial level due to oxidative processes, where a big part of phenothiazine derivatives is accumulated [50,51]. Moreover, there are studies indicating that PEG and imine units also have the ability to induce significant antioxidant activity [52,53].

**Figure 4:**
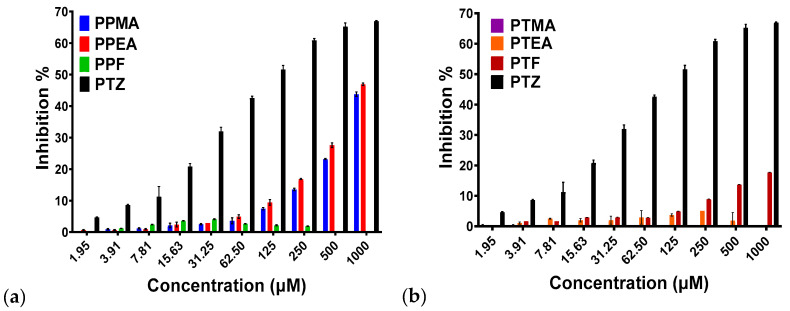
Antioxidant activity against 1,1-diphenyl-2-picryhydrazyl (DPPH) of the (**a**) PEGylated compounds, and (**b**) TEGylated compounds compared to PTZ as positive control.

In this light, the antioxidant activity of the studied phenothiazine derivatives was investigated using DPPH assay, measuring the dependence of the free radical inhibition on the concentration as highlighted by a decreasing of the absorbance of DPPH when the concentration of the compound is increasing [54,55]. The results, expressed in percentage of inhibition, are represented in Figure 4.

The spectrophotometric determination of the inhibition capacity revealed that the formylated derivatives (PTF, PPF) and TEGylated imine derivatives (PTMA, PTEA) showed very low radical scavenging capacity. However, PEGylated imine derivatives have shown radical inhibition capacity, with maximum values of 44% for PPMA and 47% for PPEA at a concentration of 1 mM yet lower than those obtained for PTZ, which displayed an inhibition of 67% at the same concentration. These data suggested that the substitution at the nitrogen atom of phenothiazine hampered its antioxidant activity, possibly due to steric hindrance effects [56]. Moreover, the quenching of the intrinsic antioxidant activity of PTZ due to the presence of a formyl unit can be attributed to the electron-acceptor effect diminishing the ability of sulfur heteroatom to interact with radical species [57]. The significant antioxidant activity of PEGylated imine derivatives compared to TEGylated ones can be attributed to the capacity of PEG to potentiate the antioxidant activity of imine units [52,58]. All in all, the obtained results indicated that PEGylated imine derivatives have a free radical scavenging potential property that could influence tumor cells’ cytotoxicity.

### 2.4. Amino Acid Binding Capacity

Amino acids play a key role in the nutritive process of normal and cancer cells [59]. Compared to a normal cell, cancer cells suffer from a lack of nutrients and the ability to synthesize their own amino acids [60]. To overcome these disadvantages, cancer cells use amino acids by taking them from surrounding areas of the body to synthesize their own proteins, enzymes and nucleotides and to obtain energy for growing [61]. One of the most important amino acids for cancer cell developing is glutamine due to its role in the uptake of essential amino acids. Also, glutamine is the primary mitochondrial substrate required for maintenance of mitochondrial membrane potential and integrity. One of the methods to stop cancer cell proliferation is the blocking of this amino acid. A rational method to do this is by a transimination process. Due to the presence of two -NH_2_ units on the glutamine molecule, an imination or transimination process could occur in the presence of the studied aldehydes and imines, respectively, favored by the dynamic character of the imine linkage in the acidic environment of tumors [43,44,45]. This process can be further advantageous by releasing the sulfonamide unit with adjuvant role in anticancer activity [62].

**Figure 5:**
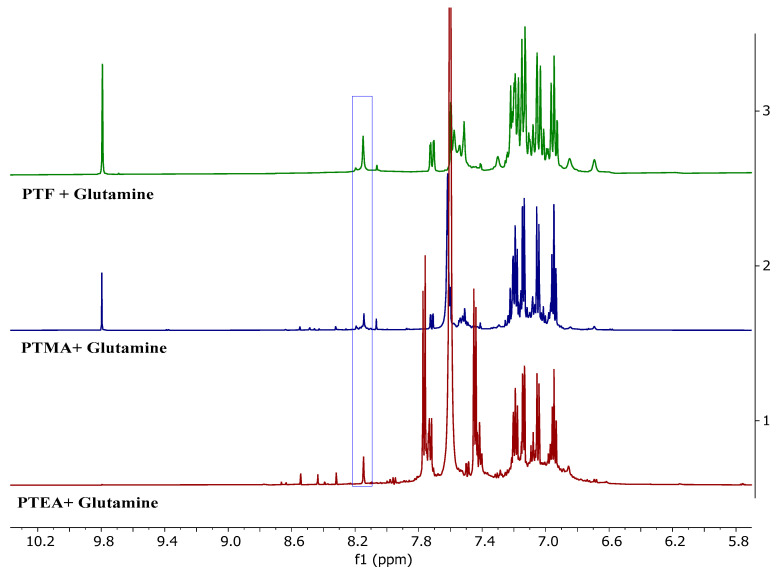
^1^H-NMR spectra of the TEGylated compounds incubated with glutamine (1/1 molar ratio, 50 mM), recorded in DMSO-d_6_.

The ability of the studied compounds to bind amino acids by imitation or transamination processes was investigated for glutamine amino acid. To do this, all compounds were incubated in the presence of glutamine for 48 h in a MEM solution to simulate in vitro tests on cancer cell lines. After incubation, the water was removed by lyophilization, and the crude solid was analyzed by NMR. All TEGylated derivatives revealed the ability to bind glutamine (Figure 5). The ^1^H-NMR spectrum of the PTF incubated with glutamine (Figure S1) showed the signal characteristic for the aldehyde proton at 9.79 ppm and the signal characteristic to imine proton at 8.15 ppm as well. Additional information about imine formation was obtained from the H,C-HSQC spectrum (Figure S2). In this type of heteronuclear bidimensional spectrum, a proton–carbon direct bond correlation signal was obtained between the proton resonating at 8.15 ppm and the carbon resonating at 160.5 ppm. This carbon chemical shift value is specific to the imine group, as we showed in a previous study [45]. 

Similarly, the ^1^H-NMR spectrum of PTMA incubated with glutamine showed the signal of the PTF and of the imine proton, indicating that the glutamine binding process took places in two steps (Figure 5). In the first step, the corresponding aldehyde is formed due to the shifting of the imination equilibrium in aqueous solution, and in the second step it is reacting with glutamine forming a new imine [45,63]. The ^1^H-NMR of PTEA incubated with glutamine indicated a different behavior. The aldehyde proton signal from 9.79 ppm was not present; only that characteristic for the new imine with glutamine was present, indicating the presence of a single-step process, which is equivalent to direct transimination (Figure 5) [64,65]. The difference between these two imine derivatives (PTMA and PTEA) is the number of methylene units between the phenothiazine core and sulfonamide moiety. PTEA has two methylene units, which allows higher molecule mobility and a lower steric hindrance, favorable for a direct transimination process [66]. 

**Figure 6:**
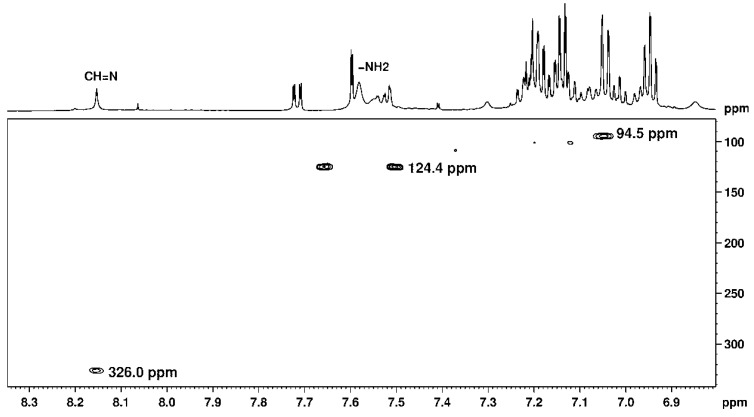
^1^H,^15^N-HMBC NMR spectrum of PTF incubated with glutamine showing the following proton-nitrogen long-range correlation: N from phenothiazine (94.5 ppm) with aromatic protons (doublet at 7.05 ppm), NH2 from the amide group (124.4 ppm) with corresponding protons (broad singlet at 7.58 ppm) and N from the imine group (326.0 ppm) with imine proton (singlet at 8.15 ppm).

Due to the fact that glutamine has two -NH_2_ units, a primary amine unit and one from the amide functional group, their ability to form imine units with the studied compounds was investigated too. It is expected that the high reactivity and the nucleophile character of the primary amine unit will prompt its preponderant reaction [67]. This hypothesis was confirmed by bidimensional long-range ^1^H,^15^N-HMBC spectra for both glutamine (Figure S3) and glutamine-based imine (Figure 6). From this type of spectrum, the chemical shift values for the two nitrogen atoms of glutamine were 39.5 ppm (primary NH_2_ group) and 111.2 ppm (amide NH_2_ group). In the case of PTF glutamine-based imine, three correlation signals were obtained, indicating the presence of three different nitrogen atoms: 94.5 ppm (N from phenothiazine), 124.4 ppm (NH2 from the amide group) and 326.0 ppm (N from the imine group) (Figure 6). The absence of the nitrogen atom from 39.5 ppm proves that the primary amine group from glutamine is involved in imine formation.

No binding activity of the PEGylated compounds was observed, most probably due to the steric hindrance of the PEG chains that have the potential to protect the imine bond [68].

**Figure 7:**
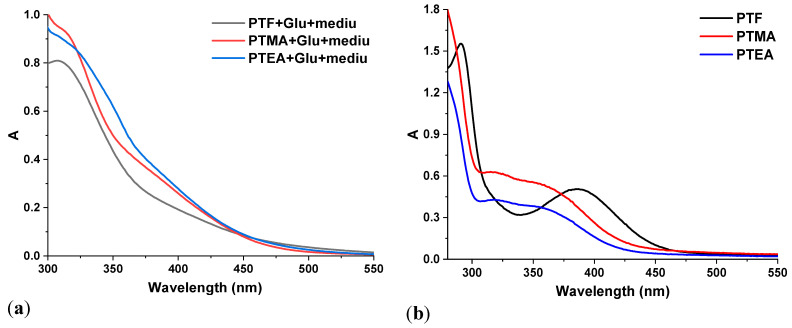
The UV-vis spectra of the (**a**) TEGylated compounds incubated with glutamine and (**b**) TEGylated compounds, recorded in DMSO.

The formation of the glutamine imines was complementarily confirmed by UV-vis spectrophotometry, which revealed the disappearance of the absorption bands characteristic of the sulfonamide moiety and of the aldehyde/imine extended conjugation (Figure 7).

### 2.5. Investigation of Farnesyltransferase Inhibition

The investigation of phenothiazine derivatives as antitumor agents revealed inhibition of the farnesyltransferase(FT) enzyme as an important factor of the antitumor mechanism [30], based on the binding of its thiol units and the coordination of Zn^2+^ or Mg^2+^ sites [29,31]. To estimate the ability of the studied compounds to inhibit the FT, their ability to bind the Zn^2+^ or Mg^2+^ ions was investigated by incubation in similar conditions as in vitro antitumor tests. The experiments indicated the ability of TEGylated derivatives to bind Mg^2+^ ions, as follows. 

**Figure 8:**
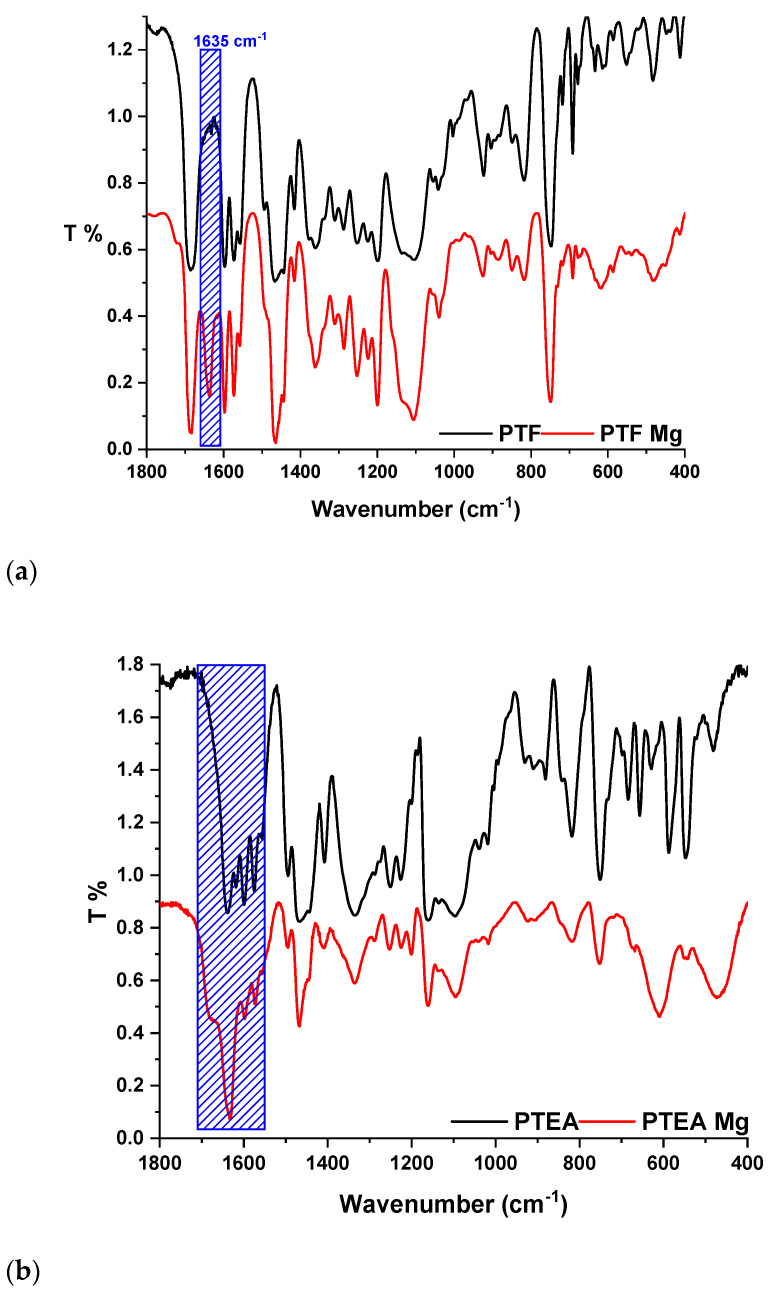
FTIR spectra of the (**a**) TEGylated aldehydes and (**b**) TEGylated imines incubated with magnesium salts compared to pristine derivatives.

The FTIR spectra of the TEGylated phenothiazine derivatives incubated with Mg^2+^ showed clear differences compared to spectra of pristine compounds, indicating bonding of the magnesium ion. Thus, the FTIR spectrum of PTF incubated with magnesium ions revealed the appearance of a new band at 1635 cm^−1^ (Figure 8a) attributed to the dative bond with magnesium. On the other hand, the band around 1635 cm^−1^ in the spectra of PTMA and PTEA (Figure 8b and Figure S4), characteristic of the imine linkage, intensified very much when incubated with magnesium ions and also attributed to the formation of dative bonds [69,70]. In addition, the spectra of the imine derivatives showed the appearance of a shoulder around 1680 cm^−1^, assigned to the formyl group formed due to the shifting of imination equilibrium in aqueous media [45].

**Figure 9:**
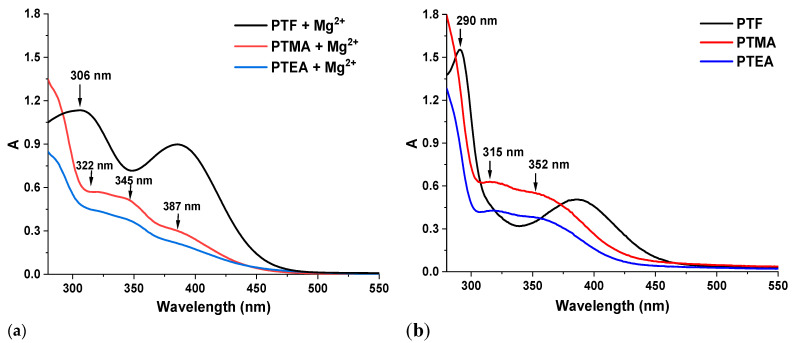
UV-vis spectra of the (**a**) TEGylated compounds incubated with magnesium salts and their (**b**) pristine counterparts.

The complexation of magnesium was also indicated by the UV-vis spectra as the bathochromic shifting of the absorption band characteristic to the conjugated system of imines was observed. This shifting to a lower energy is usually induced when around the functional groups there appears an electron host such as Mg^2+^ (Figure 9 and Figure S5a) [71,72]. In addition, the UV-vis spectra of the PTF showed a shifting of the absorption band characteristic to formyl unit, from 290 to 306 nm, suggesting that this unit acts as ligand for magnesium ion too.

Once again, similar to the transimination experiment, PEGylated compounds did not show an ability to bind Zn^2+^ or Mg^2+^ ions, indicating their inactivity as FT inhibitors.

## 3. Materials and Methods

### 3.1. Materials

Phenothiazine 98%, sodium hydride 95%, methoxy poly(ethylene glycol) (550 Da), triethylene glycol monomethyl ether 97%, phosphorus (V) oxychloride 99%, 4-(2-aminoethyl)benzenesulfonamide 99%, 4-(aminomethyl) benzenesulfonamide hydrochloride hydrate 99%, magnesium sulfate (MgSO_4_) 99.5%, magnesium chloride anhydrous (MgCl_2_) 98% and zinc acetate dihydrate (Zn(OAc)_2_ x 2H_2_O) were purchased from Sigma-Aldrich. Triethylamine 99.5% was purchased from Fluka, and N, N-dimethylformamide (DMF) 99.5%, dichloromethane (DCM) 99.5%, dichloroethane (DCE) 99% and methanol 99% were purchased from ROTH. The deuterated solvents used for the NMR analysis were purchased from Euriso-top. All reagents and solvents were used as received.

Normal human dermal fibroblasts (NHDF) cells were purchased from PromoCell, and the melanoma cell line (MeWo), osteosarcoma cell line (HOS), cervical cancer cell line (HeLa), human breast cancer cell line (MCF-7), human liver cancer cell line (HepG2), human brain glioblastoma cell line (LN-229) and glioblastoma cell line (U-118) were purchased from CLS Cell Lines Service GmbH. Eagle’s Minimal Essential Medium alpha (aMEM) and Penicillin–Streptomycin–Amphotericin B mixture were purchased from Lonza; fetal bovine serum was purchased from Sigma Aldrich; Dulbecco′s Modified Eagle′s Medium (DMEM), TrypLE Express and StemPro™ Accutase™ Cell Dissociation Reagent were purchased from Gibco; phosphate buffered saline (PBS) was purchased from Invitrogen; CellTiter 96^®^ Aqueous One Solution Cell Proliferation Assay (MTS) was purchased from Promega; and CytoOne^®^ 96-well plates were purchased from StarLab. CT26 cells were received from the LifeScience Institute, National University of Singapore.

### 3.2. Synthesis

The compounds under study were synthesized using already reported protocols [40,45], and their structure is given in Scheme 2. Briefly, phenothiazine (PTZ) has been modified by substitution at the nitrogen atom with poly(ethylene glycol) or tri(ethylene glycol) to give 10-(methoxy poly(ethylene glycol))-10H-phenothiazine (PP) and 10-(methoxy tri(ethylene glycol))-10H-phenothiazine (PT), respectively. Further, their formyl derivatives were synthetized by Vilsmeier–Haack reaction to give 10-(triethylene glycol)-10H-phenothiazine-3-carbaldehyde (PTF) and 10-(poly(ethylene glycol))-10H-phenothiazine-3-carbaldehyde (PPF). These two aldehydes were further reacted with two amines bearing benzene sulfonamide unit via microwave-assisted conjugation reaction to give four imines: 4-((((10-(triethylene glycol)-10H-phenothiazin-3-yl) methylene)amino)methyl)benzenesulfonamide (PTMA), 4-((((10-triethylene glycol)-10H-phenothiazin-3-yl)methylene)amino)ethyl)benzenesulfonamide (PTEA), 4-((((10-(poly(ethylene glycol))-10H-phenothiazin-3-yl)methylene)amino)methyl) benzenesulfonamide (PPMA) and 4-((((10-(poly(ethylene glycol))-10H-phenothiazin-3-yl)methylene)amino)ethyl)benzenesulfonamide (PPEA) (Scheme 2). The structure of the compounds and their high purity were confirmed by NMR and FT-IR spectra, as follows.

PP: ^1^H NMR (400 MHz, DMSO-d*_6_*, ppm) δ =7.19 (t, 2H), 7.14 (d, 2H), 7.05 (d, 2H), 6.94 (t, 2H), 4.04 (t, 2H), 3.74 (t, 2H), 3.49–3.47 (m, 48H), 3.43–3.41 (m, 2H), 3.24 (s, 3H); FT-IR (KBr, cm^−^): 2870 (νCH), 1593, 1570, 1460 (νC = C), 1292 (νC-N), 1110 (νC-O), 755 (δC-H).

PT: ^1^H NMR (400 MHz, CDCl_3_, ppm) δ = 7.16–7.11 (m, 4H), 6.91 (t, 4H), 4.10 (t, 2H), 3.85 (t, 2H), 3.66–3.62 (m, 6H), 3.55–3.53 (m, 2H), 3.37 (s, 3H); FT-IR (KBr, cm^−^): 2876 (νCH), 1597, 1571, 1463 (νC = C), 1291 (νC-N), 1110 (νC-O), 751 (δC-H).

PTF: ^1^H NMR (400 MHz, DMSO-d*_6_*, ppm) δ = 9.80 (s, 1H), 7.72 (dd, 1H), 7.60 (d, 1H), 7.22 (td, 1H), 7.21 (d, 1H), 7.16 (dd, 1H), 7.12 (d, 1H), 7.01 (t, 1H), 4.13 (t, 2H), 3.77 (t, 2H), 3.50–3.46 (m, 6H), 3.39–3.36 (m, 2H), 3.20 (s, 3H); FT-IR (KBr, cm^−^): 2875 (νC-H), 1679 (νC = O), 1593, 1570, 1460 (νC = C), 1292 (νC-N), 1105 (ν C-O), 751 (δC-H).

PPF: ^1^H NMR (400 MHz, DMSO-d*_6_*, ppm) δ = 9.80 (s, 1H), 7.71 (dd, 1H), 7.6 (d, 1H), 7.22 (td, 1H), 7.21 (d, 1H), 7.16 (dd, 1H), 7.12 (d, 1H), 7.01 (td, 1H), 4.13 (t, 2H), 3.78 (t, 2H), 3.51–3.47 (m, 60H), 3.24 (s, 3H); FT-IR (KBr, cm^−^): 3060 (νC-H), 2950, 2920 (νC-H), 1685 (νC = O), 1593, 1570, 1460 (νC = C), 1292 (νC-N), 1100 (νC-O), 804 (δC-H).

PTMA: ^1^H NMR (400 MHz, DMSO-d*_6_*, ppm) δ = 8.40 (s, 1H), 7.80 (d, 2H), 7.60 (dd, 1H), 7.55 (d, 1H), 7.51 (d, 2H), 7.32 (bs, 2H), 7.21 (td, 1H), 7.17 (dd, 1H), 7.13 (d, 1H), 7.09 (d, 1H), 6.98 (t, 1H), 4.80 (s, 2H), 4.10 (t, 2H), 3.77 (t, 2H), 3.56–3.54 (m, 2H), 3.51–3.47 (m, 4H), 3.40–3.37 (m, 2H), 3.20 (s, 3H); FT-IR (KBr, cm^−^): 3333, 3239 (νN-H), 2932, 2875 (νC-H), 1638 (νC = N), 1598, 1570, 1466 (νC = C), 1335, 1153 (νS = O), 1292 (νC-N), 1096 (νC-O), 748 (δC-H).

PTEA: ^1^H NMR (400 MHz, DMSO-d*_6_*, ppm) δ = 8.15 (s, 1H), 7.72 (d, 2H), 7.49 (dd, 1H), 7.45 (d, 1H), 7.42 (d, 2H), 7.27 (bs, 2H), 7.20 (td, 1H) 7.15 (dd, 1H), 7.08 (d, 1H), 7.07 (d, 1H), 6.97 (t, 1H), 4.07 (t, 2H), 3.78 (t, 2H), 3.75 (t, 2H), 3.55–3.36 (m, 8H), 3.20 (s, 3H), 2.98 (t, 2H); FT-IR (KBr, cm^−^): 3317, 3228 (νN-H), 2932, 2875 (νC-H), 1638 (νC = N), 1593, 1570, 1466 (νC = C), 1335, 1153 (νS = O), 1292 (νC-N), 1096 (νC-O), 748 (δC-H).

PPMA: ^1^H NMR (400 MHz, DMSO-d*_6_*, ppm) δ = 8.41 (s, 1H), 7.80 (d, 2H), 7.60 (dd, 1H), 7.55 (d, 1H), 7.51 (d, 2H), 7.33 (bs, 2H), 7.21 (td, 1H), 7.16 (dd, 1H), 7.13 (d, 1H), 7.09 (d, 1H), 6.98 (t, 1H), 4.80 (s, 2H), 4.10 (t, 2H), 3.77 (t, 2H), 3.47–3.52 (m, 70H), 3.24 (s, 3H); FT-IR (KBr, cm^−^): 3277, 3228 (νN-H), 2875 (νCH), 1642 (νC = N), 1598, 1570, 1472 (νC = C), 1349, 1161 (νS = O), 1292 (νC-N), 1100 (νC-O), 754 (δC-H).

PPEA: ^1^H NMR (400 MHz, DMSO-d*_6_*, ppm) δ = 8.16 (s, 1H), 7.72 (d, 2H), 7.49 (d, 1H), 7.46 (s, 1H), 7.43 (d, 2H), 7.28 (bs, 2H), 7.20 (t, 1H), 7.15 (d, 1H), 7.08 (d, 1H), 7.07 (d, 1H), 6.98 (t, 1H), 4.07 (t, 2H), 3.79–3.74 (m, 4H), 3.50 (m, 60H), 3.23 (s, 3H), 2.98 (t, 2H); FT-IR (KBr, cm^−^): 3287, 3237 (νN-H), 2920, 2875 (νC-H), 1642 (νC = N), 1598, 1570, 1472 (νC = C), 1341, 1163 (νS = O), 1292 (νC-N), 1100 (νC-O), 754 (δ C-H).

### 3.3. Equipment and Methods

The NMR spectra for phenothiazine formylated and pegylated derivatives were recorded on 400 MHz Bruker Avance Neo spectrometer equipped with a 5 mm 4-nuclei direct detection z-gradient probe. The NMR spectra for glutamine and glutamine-based imines were recorded on a Bruker Avance NEO 600 MHz spectrometer equipped with a 5 mm multinuclear inverse detection z-gradient probe, at room temperature. Proton spectra were acquired with 64 scans in order to overcome the low concentration of imine groups. ^1^H,^13^C-HSQC (Heteronuclear Single Quantum Coherence) and ^1^H,^15^N-HMBC (Heteronuclear Multiple Bond Correlation) NMR experiments were recorded using standard pulse sequence delivered by Bruker with TopSpin 4.0.8 spectrometer control and processing software. Proton chemical shifts were reported as δ values (ppm) relative to the residual solvent signal. Carbon and nitrogen chemical shifts were obtained as 1D projections from the corresponding bidimensional spectra.

Infrared spectra were recorded on a FTIR Bruker Vertex 70 Spectrometer, at room temperature, using KBr pellets.

The absorbance for CellTiter 96^®^ Aqueous One Solution Cell Proliferation Assay (MTS) and for DPPH inhibition studies was measured using a FLUOstar Omega Filter-based multimode microplate reader from BMG LABTECH.

Solubility tests were performed to establish the maximum concentration of the compounds that might be used for in vitro tests by dissolving 0.003 mmol of compound in 1mL of water (H_2_O) with 1% of DMSO. The amount of solvent was increased until the compound was completely dissolved. The volume of solvent corresponding to the solving point was used to calculate the concentration. The TEGylated compounds were soluble under 0.3 mM (PTF) and 0.2 mM (PTMA, PTEA), and PEGylated compounds were soluble at to a concentration of 2 mM.

Cell culture: NHDF, HeLa, HOS, HepG2, MCF7, MeWo and CT-26 cells were cultivated in complete Eagle’s Minimal Essential Medium alpha (aMEM) containing a 1% Penicillin–Streptomycin–Amphotericin B mixture and a 10% fetal bovine serum under 5% CO2 humidified atmosphere at 37 °C. LN-229 cells were cultivated in DMEM with a 1% Penicillin–Streptomycin–Amphotericin B mixture and a 5% fetal bovine serum. U-118 cells were cultivated in DMEM with with a 1% Penicillin–Streptomycin–Amphotericin B mixture and a 10% fetal bovine serum. StemPro™ Accutase™ Cell Dissociation Reagent was used for passaging MeWo and U-118 cells, and TrypLE Express was used for all other cell lines. 

The cells were maintained in culture dishes in MEM alpha with a 10% fetal bovine serum and a 1% antibiotic–antimycotic mixture until they reached subconfluency in a humid atmosphere and with 5% carbon dioxide.

Preparation of the solutions for in vitro biologic testing: Amounts of 11.2 mg PTF, 22.7 mg of PPF, 16.2 mg PTMA, 16.6 mg PTEA, 27.8 mg PPMA, 28.2 mg PPEA and 6 mg PTZ were dissolved in 1 mL DMSO to obtain 30 mM stock solutions. The working solutions were prepared by diluting the stock solutions with complete medium so that the final concentration of DMSO in the cell culture was 1%. The working solutions were used for incubation. Control cells were treated only with complete cell culture medium containing 1% DMSO.

Cytotoxicity MTS assay: In order to perform the MTS assay, cells were placed in culture-treated 96-well plates at densities of 5 × 10^3^ cells/well (NHDF and CT-26), 7 × 10^3^ cells/well (MCF7) and 10 × 10^3^ cells/well (HOS, HeLa, HepG2, MeWo, LN-229, U-118) in 100 μL of a MEM medium/well and allowed to adhere for 24 h. After 24 h, the culture medium was replaced with the tested compound solutions at various concentrations, and the plates were incubated for another 48 h. Before the final reading, 20 μL CellTiter 96^®^ Aqueous One Solution/well was added, and the plates were incubated for 1–4 h. Finally, the absorbance was recorded with a microplate reader at λ = 490 nm. The relative cell viability was expressed as a percentage of the viability of control, and the half maximal inhibitory concentration (IC_50_) was determined from the graphical representation of the cell viability vs. concentration. Graphical data were expressed as means ± standard error of the mean (S.E.M.). Statistical analysis was made with two-way ANOVA using Tukey’s multiple comparisons test. The difference was considered significant when *p* < 0.05. Data analysis was performed with GraphPad Prism software version 7.00 for Windows.

A selectivity analysis was performed using the NHDF line as a control to determine the cytotoxic selectivity of PP and PPO. The selectivity index (SI) was calculated with the following Equation (1) [73]:

SI = IC_50_ normal cell line/IC_50_ tumor cell line (1)

Ex Vivo Radical Scavenging Activity: In order to evaluate antioxidant activity of the studied compounds, a bleaching method of the 1,1-diphenyl-2-picryl hydrazyl (DPPH) radical was used. Briefly, a stock solution of 2 mM of each compound in ethanol was prepared and then diluted to obtain 10 different concentrations from 1 mM to 0.0019 mM using a 96-well plate. The diluted solutions with different concentrations were mixed with an equal volume of ethanolic DPPH solution (0.05 mg/mL), and the mixtures were incubated in the dark at room temperature (37 °C) for 1 h. After incubation, the absorbance was read at 517 nm. Inhibition percentage was calculated using Equation (2), where Ac is the absorbance of the control sample (DPPH) and As is the absorbance of the mixtures. All measurements were made in triplicate.

I % = (Ac-As)/Ac × 100 (2)

Glutamine transimination protocol: An amount of 0.05 mmol of each compound (PPF, PTF, PPMA, PPEA, PTMA, PTEA) was mixed with glutamine in a 1/1 molar ratio in 1 mL MEM medium. The mixtures were incubated at 37 °C for 48 h in order to simulate the in vitro conditions of antitumor determinations. After the incubation, all the samples were lyophilized, dissolved in DMSO-d*_6_* and subjected to NMR spectroscopy analysis. The samples that indicated the imine formation were further investigated by recording the ^1^H, ^15^N HMBC and ^1^H, ^13^C HSQC NMR spectra. The ^1^H-NMR and ^1^H, ^15^N HMBC NMR spectra of the glutamine dissolved in a mixture of 9/1 H_2_O/D_2_O were also provided as reference in order to determine which nitrogen was involved in the imine linkage. After the NMR studies, the same samples were diluted with DMSO to obtain a solution of 0.33 mM in order to provide their UV-vis spectra. 

Complex formation protocol: In the first stage, all studied compounds (PPF, PTF, PPMA, PPEA, PTMA, PTEA) and MgCl_2_ and Zn(OAc)_2_x2H_2_O were dissolved in ultrapure water to obtain 10–3 M solutions. After that, the solutions were combined in a 2/1 molar ratio of the compounds/metallic salt, leading to 12 mixtures—PPF + Zn^2+^, PTF + Zn^2+^, PPMA + Zn^2+^, PPEA + Zn^2+^, PTMA + Zn^2+^, PTEA + Zn^2+^, PPF + Mg^2+^, PTF + Mg^2+^, PPMA + Mg^2+^, PPEA + Mg^2+^, PTMA + Mg^2+^ and PTEA + Mg^2+^—that were magnetically stirred for 30 min and then incubated for 2 days at 37 °C in similar conditions with in vitro antitumor tests. After that, all the solutions were lyophilized, and the solid samples obtained were subjected to the FTIR spectroscopy measurements. Then, the mixtures that indicated complex formation were dissolved in DMSO, and their UV-vis spectra were recorded.

## 4. Conclusions

The investigation of the in vitro antitumor activity of some PEGylated and TEGylated phenothiazine derivatives functionalized with sulfonamide unit via dynamic imine bonds compared to their formyl counterparts revealed interesting aspects regarding the structure–activity relationship. Thus, the TEGylated derivatives showed high selectivity and increased specificity on HeLa cells with a IC_50_ around 0.015 mM. The replacing of TEG with PEG was followed by an increased cytotoxicity and also a modification of the specificity of the PEGylated imine derivatives for HepG2 and CT26 cells, reaching IC_50_ values of 0.04 mM and 0.029 mM, respectively. The cytotoxic activity was improved by the presence of the sulfonamide unit and the ethylene unit as well. The investigation of a possible antitumor mechanism indicated that different building blocks induced different functionalities that can be responsible for different antitumor activities. Thus, the phenothiazine derivatives containing (i) PEG and imine units showed antioxidant activity being potent to reduce the quantity of free radicals from cancer cell environment, while (ii) TEG and imine units showed the potential to interact with amino acids by imination or transimination processes, and (iii) TEGylated derivatives showed the ability to bind magnesium ions, emphasizing the capacity to inhibit farnesyltransferase. Furthermore, the PEG chain induced better solubility pointing for the improvement of antitumor activity due to bioavailability increases. It was concluded that different building blocks and combinations of building blocks endowed the phenothiazine derivatives with different functionalities that governed their antitumor activity. Compared to traditional antitumor drugs, such as doxorubicin and 5-Fluorouracil, the studied compounds presented a greater selectivity index, pointing to the diminishing of side effects. In this light, the new design can be a good starting point for developing a new generation of antitumor drugs.

## Data Availability

Not applicable.

## Supplementary Materials

The following supporting information can be downloaded at https://www.mdpi.com/article/10.3390/ijms24065449/s1. References [74,75,76,77,78,79,80,81,82,83,84,85,86,87,88,89,90,91,92] are cited in the Supplementary Materials.
