# Supplementary material for: Antitumor Activity of PEGylated and TEGylated Phenothiazine Derivatives: Structure–Activity Relationship
Source: Int J Mol Sci. 2023 Mar 13;24(6):5449. doi: 10.3390/ijms24065449 (PMC10049495; doi:10.3390/ijms24065449)
Supplement: Supplementary file 1 [file ijms-24-05449-s001.zip › ijms-2191695-supplementary.pdf]

Supporting Information

for

Antitumor Activity of PEGylated and TEGylated Phenothiazine Derivatives: Structure–Activity Relationship

Sandu Cibotaru, Andreea-Isabela Sandu, Alina Nicolescu and Luminita Marin *
"Petru Poni" Institute of Macromolecular Chemistry, 700487 Iasi, Romania
* Correspondence: lmarin@icmpp.ro

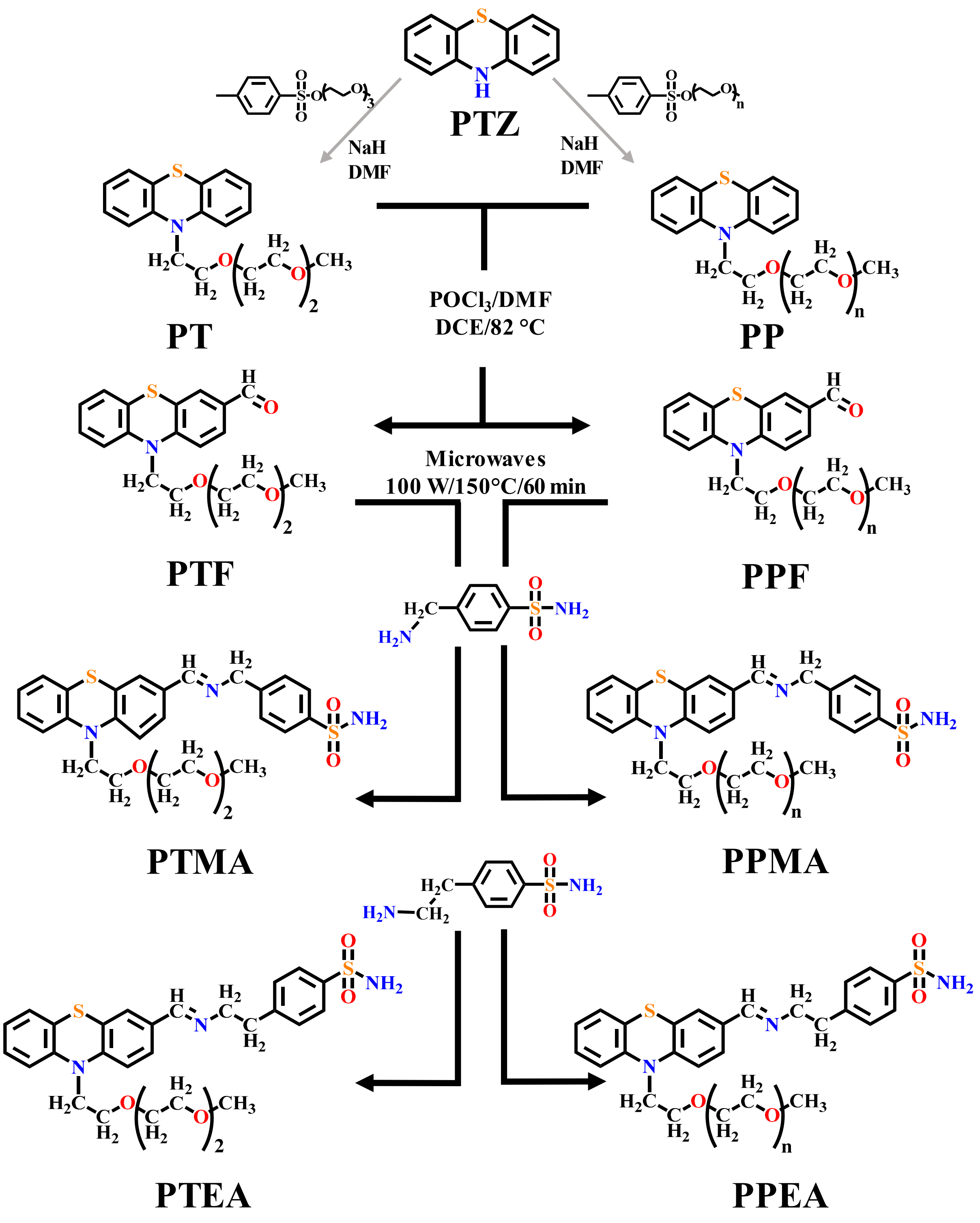

Sheme S1. The synthesis pathway of the studied compounds

Table S1. Half maximal inhibition concentration (IC_{50}) of TEGylated and PEGylated derivatives on different tumor lines and a normal cell line. Cells were treated with various concentrations of PEGylated phenothiazines for 48 h. Cells viability was determined by MTS assay to obtain IC_{50} values. The yellow marked values presented a higher concentration than the last used for experiment.

Code / Cell Line	PTZ	PPF	PTF	PTMA	PTEA	PPMA	PPEA
**NHDF**	0.175	>0.300	0.150	0.200	0.107	0.150	0.108
**CT-26**	0.186	0.139	0.063	0.107	0.080	0.040	0.029
**HeLa**	0.240	>0.300	0.015	0.029	0.015	0.116	0.086
**HepG2**	0.073	0.106	0.050	0.083	0.085	0.054	0.040
**HOS**	>0.300	>0.300	>0.300	>0.300	>0.300	>0.300	>0.300
**LN229**	>0.300	>0.300	0.186	0.219	0.183	0.239	0.109
**MCF-7**	0.188	0.270	0.108	0.141	0.138	0.090	0.090
**MeWo**	>0.300	0.235	0.147	0.287	0.239	0.104	0.068
**U-118**	>0.300	>0.300	0.190	>0.300	0.170	0.092	0.100

Table S2. Selectivity index of TEGylated and PEGylated derivcatives on tested tumor cell lines

Selectivity index (SI)							
	PTZ	PTF	PPF	PTMA	PTEA	PPMA	PPEA
CT-26	-	2.38	-	2	1.25	3.75	3.44
HeLa	-	10	-	6.89	6.66	1.36	1.25
HepG2	2.42	3	-	2.5	1.25	3	2.5
HOS	-	-	-	-	-	-	-
LN229	-	-	-	-	-	-	-
MCF7	-	1.5	-	1.42	-	1.66	1.11
MeWo	-	-	-	-	-	1.5	1.66
U118MG	-	-	-	-	-	1.66	-

Table S3. IC_{50} values for different tumor cell lines, of two therapeutic antitumor drugs

Drug	Cell Line	IC_{50} (μM)	Reference
Doxorubicin	HepG2	1.03	[74]
	HeLa	0.18	[75]
	MCF-7	0.07	
	CT-26	10	[76]
	MeWo	88	[77]
	NHDF	0.137	[78]
5-Florouracil	HepG2	253	[79]
	HeLa	676.9	
	MCF-7	12.7	[80]
	CT-26	300	[81]
	MeWo	61.5	[82]
	NHDF	25	[78]

Table S4. Half maximal inhibition concentration (IC_{50}) of other phenothiazine-based compounds determined on different tumor lines

Drug	Cell Line	IC_{50} (μM)	Reference
10-{[1-(4-Chlorophenyl)-1H-1,2,3-triazol-4-yl]methyl}-2-{trifluoromethyl}-10H-phenothiazine	MCF-7	29.6	[80]
10-{[1-(3,4,5-Trimethoxybenzyl)-1H-1,2,3-triazol-4-yl]methyl}-10H-phenothiazine		0.8	
3-([1,2,4]Triazolo[4,3-a]pyridin-3-yl)-10-ethyl-7-(4-methoxyphenyl)-10H-phenothiazine		57.2	[25]
3-([1,2,4]Triazolo[4,3-a]pyridin-3-yl)-10-ethyl-7-phenyl-10H-phenothiazine		10.2	
Trifluoperazine		11.3	[83]
Thioridazine		12.9	
1-[(10H-Phenothiazin-3-yl)carbonyl]-3-acetyl-7-methylindolizine		>10	[84]
1-[(10H-Phenothiazin-3-yl)carbonyl]-3-acetyl-6,8-dimethylindolizine		>10	
1-[(10H-Phenothiazin-3-yl)carbonyl]-3-acetyl-7-methoxyindolizine		>10	
Thioridazine	U87-MG	12.4	[85]
Prochlorperazine		0.97	[86]
Perphenazine		6.8	
Fluphenazine	CBM8401	10.0	
thioridazine	HepG2	45.5	[87]
10-((1-(4-Chlorophenyl)-1H-1,2,3-triazol-4-yl)methyl)-10Hphenothiazine		1.3	[88]
10-((1-(4-Methoxyphenyl)-1H-1,2,3-triazol-4-yl)methyl)-10Hphenothiazine (7f)		15.0	
1-Phenethylamino-3-(10H-phenothiazin-10-yl)-2-propanol	HeLa	10.0	[89]
N-alkyl-3-formylphenothiazine		57.69	[90]
Chlorpromazine	Colo205	10.0	[91]
Fluphenazine		7.18	[92]

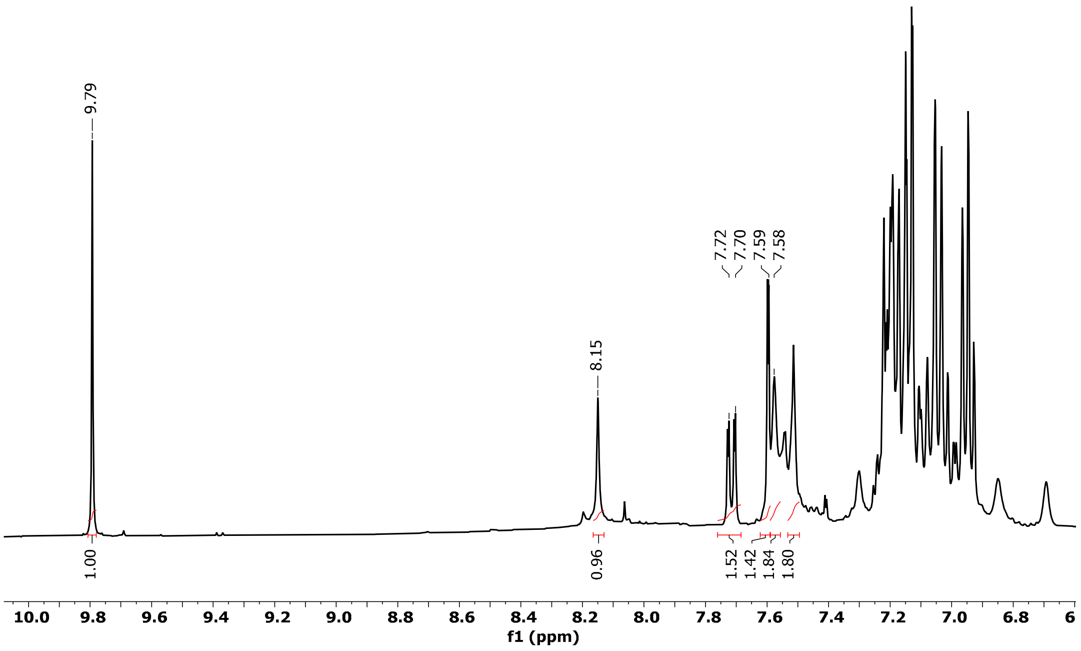

Figure S1. The ^{1}H-NMR spectrum for the PTF-glutamine mixture, recorded in DMSO-d_6, at 600 MHz.

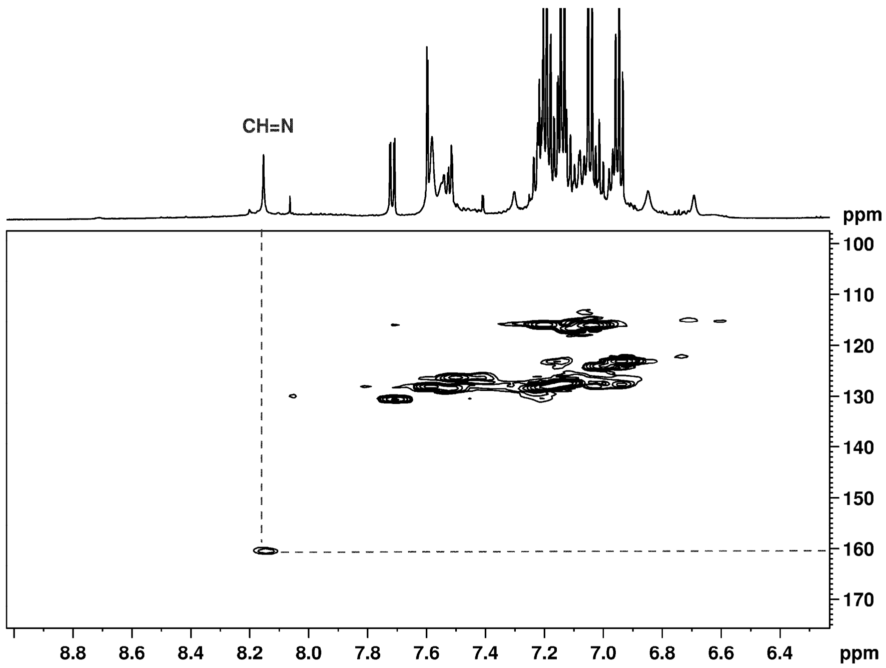

Figure S2. The ^{1}H,^{13}C-HSQC NMR spectrum for the PTF-glutamine mixture, showing the direct bond correlation signal between proton from 8.15 ppm and carbon from 160.5 ppm, as prove for amine group formation.

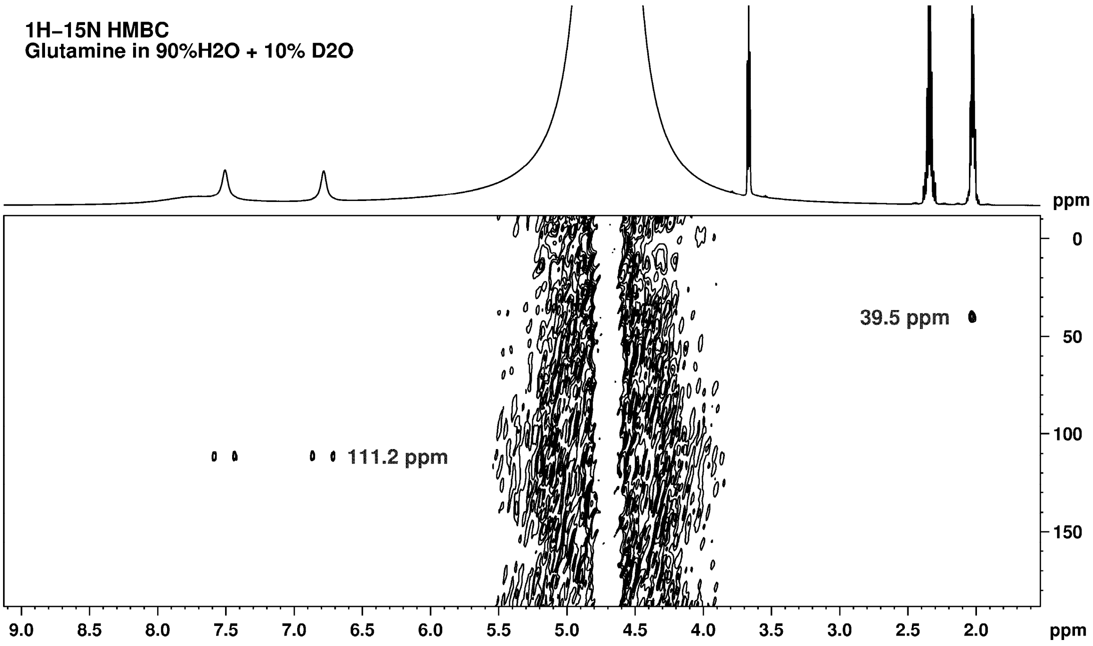

Figure S3. The ^{1}H,^{15}N-HMBC NMR spectrum for glutamine, recorded in 9/1 H_2O/D_2O mixture, showing proton-nitrogen correlation signals at 39.5 ppm (primary amine group) and 111.2 ppm (amide group).

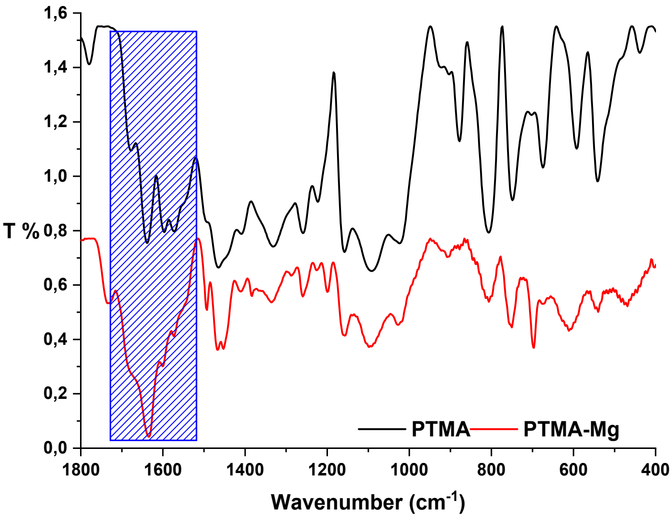

Figure S4. FTIR spectra of the PTMA incubated with magnesium salts compared to pristine derivatives

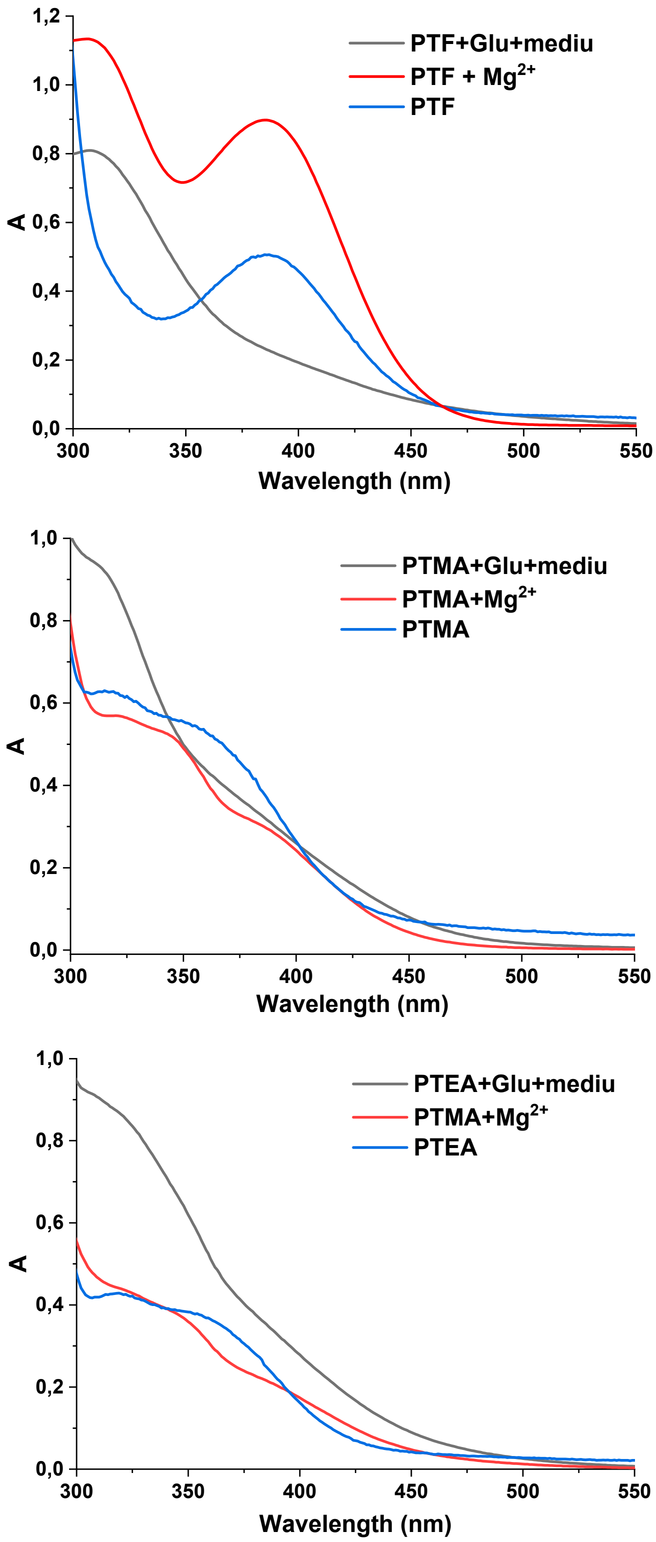

Figure S5. The comparative UV-vis spectra of the TEGylated derivatives with their mixture with glutamine and with Mg^{2+}: a) PTF, b) PTMA, c) PTEA

Funding

This research was funded by a grant from the Romanian Ministry of Education and Research, CNCS - UEFISCDI, project number PCE2/2021 within PNCDI III.

Rferences

Dubbelboer, I.R.; Pavlovic, N.; Heindryckx, F.; Sjögren, E.; Lennernäs, H. Liver Cancer Cell Lines Treated with Doxorubicin under Normoxia and Hypoxia: Cell Viability and Oncologic Protein Profile. *Cancers (Basel)* **2019**, *11*, 1024, doi:10.3390/cancers11071024.

Khan, F.M.; Saleh, E.; Alawadhi, H.; Harati, R.; Zimmermann, W.-H.; El-Awady, R. Inhibition of Exosome Release by Ketotifen Enhances Sensitivity of Cancer Cells to Doxorubicin. *Cancer Biol Ther* **2018**, *19*, 25–33, doi:10.1080/15384047.2017.1394544.

Jeong, Y.-I.; Park, H.-K.; Lee, S.J.; Oh, J.-S.; Lee, S.-G.; Lee, H.C.; Hwan Kang, D. Enzyme-Responsive Doxorubicin Release from Dendrimer Nanoparticles for Anticancer Drug Delivery. *Int J Nanomedicine* **2015**, 5489, doi:10.2147/IJN.S87145.

Durand, N.; Simsir, M.; Signetti, L.; Labbal, F.; Ballotti, R.; Mus-Veteau, I. Methiothepin Increases Chemotherapy Efficacy against Resistant Melanoma Cells. *Molecules* **2021**, *26*, 1867, doi:10.3390/molecules26071867.

Zych, D.; Slodek, A.; Krompiec, S.; Malarz, K.; Mrozek-Wilczkiewicz, A.; Musiol, R. 4′-Phenyl-2,2′:6′,2″-Terpyridine Derivatives Containing 1-Substituted-2,3-Triazole Ring: Synthesis, Characterization and Anticancer Activity. *ChemistrySelect* **2018**, *3*, 7009–7017, doi:10.1002/slct.201801204.

Yuan, F.; Qin, X.; Zhou, D.; Xiang, Q.-Y.; Wang, M.-T.; Zhang, Z.-R.; Huang, Y. In Vitro Cytotoxicity, in Vivo Biodistribution and Antitumor Activity of HPMA Copolymer–5-Fluorouracil Conjugates. *European Journal of Pharmaceutics and Biopharmaceutics* **2008**, *70*, 770–776, doi:10.1016/j.ejpb.2008.06.028.

Zhang, J.-X.; Guo, J.-M.; Zhang, T.-T.; Lin, H.-J.; Qi, N.-S.; Li, Z.-G.; Zhou, J.-C.; Zhang, Z.-Z. Antiproliferative Phenothiazine Hybrids as Novel Apoptosis Inducers against MCF-7 Breast Cancer. *Molecules* **2018**, *23*, 1288, doi:10.3390/molecules23061288.

Pangeni, R.; Choi, S.W.; Jeon, O.-C.; Byun, Y.; Park, J.W. Multiple Nanoemulsion System for an Oral Combinational Delivery of Oxaliplatin and 5-Fluorouracil: Preparation and in Vivo Evaluation. *Int J Nanomedicine* **2016**, *Volume 11*, 6379–6399, doi:10.2147/IJN.S121114.

Freitas, L.B.O.; Boaventura, M.A.D.; Santos, W.L.; Stehmann, J.R.; Junior, D.D.; Lopes, M.T.P.; Magalhães, T.F.F.; da Silva, D.L.; de Resende, M.A. Allelopathic, Cytotoxic and Antifungic Activities of New Dihydrophenanthrenes and Other Constituents of Leaves and Roots Extracts of Banisteriopsis Anisandra (Malpighiaceae). *Phytochem Lett* **2015**, *12*, 9–16, doi:10.1016/j.phytol.2015.02.006.

Sachdeva, T.; Low, M.L.; Mai, C.; Cheong, S.L.; Liew, Y.K.; Milton, M.D. Design, Synthesis and Characterisation of Novel Phenothiazine-Based Triazolopyridine Derivatives: Evaluation of Anti-Breast Cancer Activity on Human Breast Carcinoma. *ChemistrySelect* **2019**, *4*, 12701–12707, doi:10.1002/slct.201903203.

Gao, Y.; Sun, T.-Y.; Bai, W.-F.; Bai, C.-G. Design, Synthesis and Evaluation of Novel Phenothiazine Derivatives as Inhibitors of Breast Cancer Stem Cells. *Eur J Med Chem* **2019**, *183*, 111692, doi:10.1016/j.ejmech.2019.111692.

Moise, I.-M.; Bîcu, E.; Farce, A.; Dubois, J.; Ghinet, A. Indolizine-Phenothiazine Hybrids as the First Dual Inhibitors of Tubulin Polymerization and Farnesyltransferase with Synergistic Antitumor Activity. *Bioorg Chem* **2020**, *103*, 104184, doi:10.1016/j.bioorg.2020.104184.

Chu, C.-W.; Ko, H.-J.; Chou, C.-H.; Cheng, T.-S.; Cheng, H.-W.; Liang, Y.-H.; Lai, Y.-L.; Lin, C.-Y.; Wang, C.; Loh, J.-K.; et al. Thioridazine Enhances P62-Mediated Autophagy and Apoptosis Through Wnt/β-Catenin Signaling Pathway in Glioma Cells. *Int J Mol Sci* **2019**, *20*, 473, doi:10.3390/ijms20030473.

Xu, F.; Xia, Y.; Feng, Z.; Lin, W.; Xue, Q.; Jiang, J.; Yu, X.; Peng, C.; Luo, M.; Yang, Y.; et al. Repositioning Antipsychotic Fluphenazine Hydrochloride for Treating Triple Negative Breast Cancer with Brain Metastases and Lung Metastases. *Am J Cancer Res* **2019**, *9*, 459–478.

de Faria, P.A.; Bettanin, F.; Cunha, R.L.O.R.; Paredes-Gamero, E.J.; Homem-de-Mello, P.; Nantes, I.L.; Rodrigues, T. Cytotoxicity of Phenothiazine Derivatives Associated with Mitochondrial Dysfunction: A Structure-Activity Investigation. *Toxicology* **2015**, *330*, 44–54, doi:10.1016/j.tox.2015.02.004.

Ma, X.-H.; Liu, N.; Lu, J.-L.; Zhao, J.; Zhang, X.-J. Design, Synthesis and Antiproliferative Activity of Novel Phenothiazine-1,2,3-Triazole Analogues. *J Chem Res* **2017**, *41*, 696–698, doi:10.3184/174751917X15122516000140.

Okumura, H.; Nakazawa, J.; Tsuganezawa, K.; Usui, T.; Osada, H.; Matsumoto, T.; Tanaka, A.; Yokoyama, S. Phenothiazine and Carbazole-Related Compounds Inhibit Mitotic Kinesin Eg5 and Trigger Apoptosis in Transformed Culture Cells. *Toxicol Lett* **2006**, *166*, 44–52, doi:10.1016/j.toxlet.2006.05.011.

Venkatesan, K.; Satyanarayana, V.S. v.; Sivakumar, A. Synthesis and Biological Evaluation of Novel Phenothiazine Derivatives as Potential Antitumor Agents. *Polycycl Aromat Compd* **2023**, *43*, 850–859, doi:10.1080/10406638.2021.2021254.

Gutierrez, A.; Pan, L.; Groen, R.W.J.; Baleydier, F.; Kentsis, A.; Marineau, J.; Grebliunaite, R.; Kozakewich, E.; Reed, C.; Pflumio, F.; et al. Phenothiazines Induce PP2A-Mediated Apoptosis in T Cell Acute Lymphoblastic Leukemia. *Journal of Clinical Investigation* **2014**, *124*, 644–655, doi:10.1172/JCI65093.

Choi, J.H.; Yang, Y.R.; Lee, S.K.; Kim, S.-H.; Kim, Y.-H.; Cha, J.-Y.; Oh, S.-W.; Ha, J.-R.; Ryu, S.H.; Suh, P.-G. Potential Inhibition of PDK1/Akt Signaling by Phenothiazines Suppresses Cancer Cell Proliferation and Survival. *Ann N Y Acad Sci* **2008**, *1138*, 393–403, doi:10.1196/annals.1414.041.
